# Expression and Biological Activity of the Cystine Knot Bioinsecticide PA1b (Pea Albumin 1 Subunit b)

**DOI:** 10.1371/journal.pone.0081619

**Published:** 2013-12-11

**Authors:** Vanessa Eyraud, Lamis Karaki, Isabelle Rahioui, Catherine Sivignon, Pedro Da Silva, Yvan Rahbé, Corinne Royer, Frédéric Gressent

**Affiliations:** BF2I (Biologie Fonctionnelle, Insectes et Interactions), INRA - INSA-Lyon, Villeurbanne, France; University of South Florida College of Medicine, United States of America

## Abstract

The PA1b (Pea Albumin 1, subunit b) peptide is an entomotoxin extract from Legume seeds with lethal activity on several insect pests, such as mosquitoes, some aphids and cereal weevils. This 37 amino-acid cysteine-rich peptide has been, until now, obtained by biochemical purification or chemical synthesis. In this paper, we present our results for the transient production of the peptide in *Nicotiana benthamiana* by agro-infiltration, with a yield of about 35 µg/g of fresh leaves and maximum production 8 days after infiltration. PA1b is part of the *PA1* gene which, after post-translational modifications, encodes two peptides (PA1b and PA1a). We show that transforming tobacco with the PA1b cDNA alone does not result in production of the toxin and, in fact, the entire cDNA is necessary, raising the question of the role of PA1a. We constructed a PA1-cassette, allowing for the quick “cut/paste” of different PA1b mutants within a conserved *PA1* cDNA. This cassette enabled us to produce the six isoforms of PA1b which exist in pea seeds. Biological tests revealed that all the isoforms display similar activity, with the exception of one which is inactive. The lack of activity in this isoform led us to conclude that the amphiphilic nature of the peptide is necessary for activity. The possible applications of this expression system for other cysteine-rich biomolecules are discussed.

## Introduction

Chemical pesticides are increasingly used around the world but they are also increasingly stigmatized because of their persistence and their toxicity to non-target organisms. Crop protection against two very important pests, namely cereal weevils and aphids, is currently carried out almost exclusively by chemical treatments. A few alternative methods do exist to combat these insects, but they are either much less effective or prohibitively expensive, compared with chemical control.

Finding new plant-derived molecules, which have less impact on the environment, is a major challenge in order to develop biopesticides for sustainable and healthy agriculture. The PA1b (Pea Albumin 1, subunit b) peptide is an entomotoxin extract from pea seeds and, more generally, from Legume seeds [1]. PA1b is one of the few orally active peptide toxins currently known. It displays outstanding insecticidal activity against certain insects, such as grain weevils (genus *Sitophilus*) and the mosquitoes *Culex pipiens* and *Aedes aegyptii* (for review see [2].

**Figure 1:**
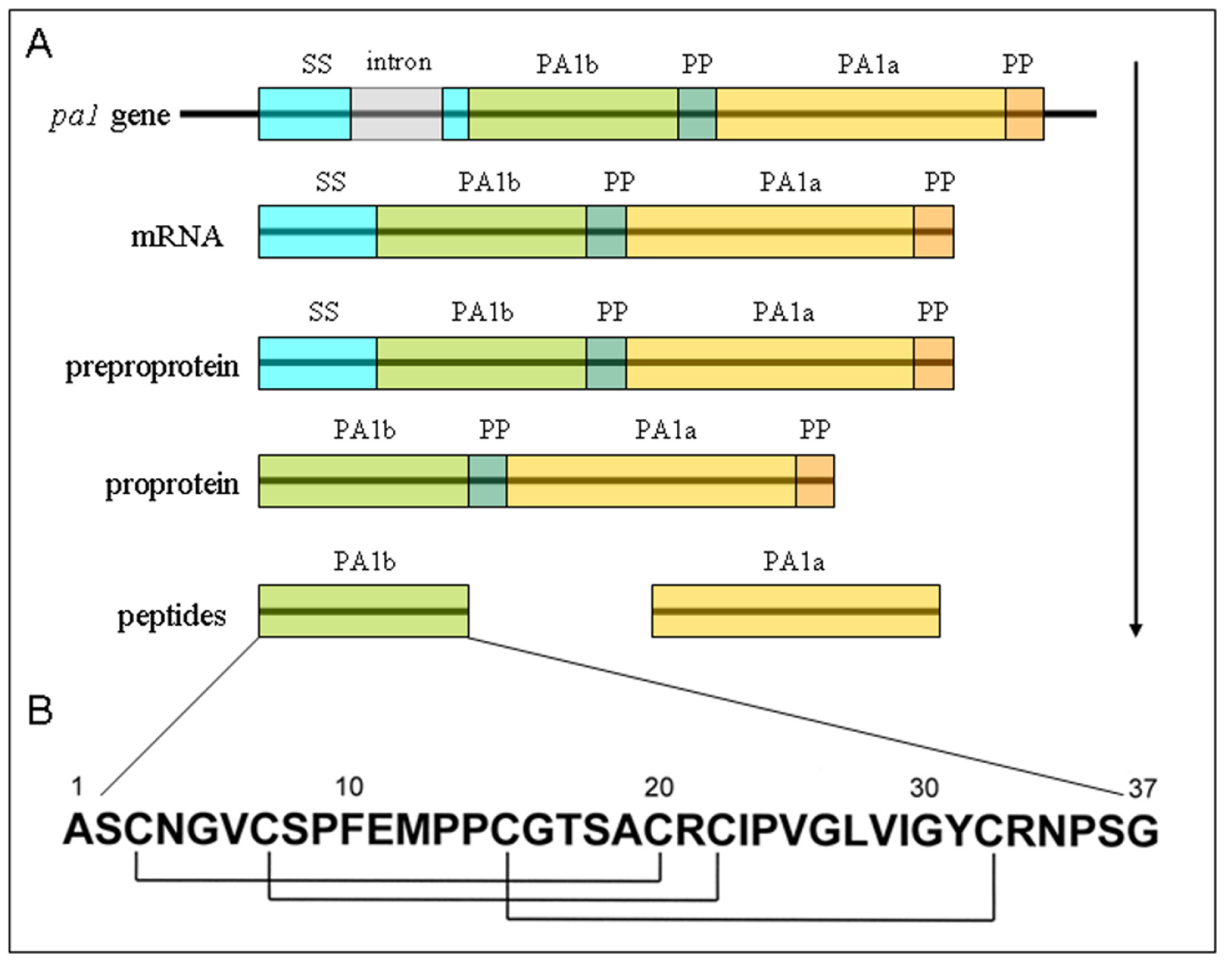
The structure of the pea albumin 1 gene (*PA1* gene). **A.** The *PA1* gene is transcribed as a single mRNA encoding the preproprotein PA1 (molecular weight 13.9 kDa; 130 amino-acids). After cleavage of its signal sequence (SS), the proprotein precursor (11.2 kDa, 104 aa) is endoproteolytically cleaved to yield two peptides which, after removal of two propeptides (PP), represent the mature forms of PA1a (6 kDa, 53 aa) and PA1b (3.8 kDa, 37 aa) [3]. **B.** PA1b primary structure. The cysteine residue pairing is shown below [4].

PA1b consists of 37 amino acids, including six cysteines forming three intramolecular disulfide bridges (Figure 1B). The structure of the pea albumin 1 gene (*PA1* gene) is depicted in Figure 1A. The *PA1* gene is transcribed as a single mRNA encoding the 13-kDa preproprotein PA1. After cleavage of its signal peptide, the proprotein precursor is endoproteolytically cleaved to yield two peptides which represent the mature forms of PA1a (6 kDa, 53 aa) and PA1b (3.8 kDa, 37 aa) [3].

The three-dimensional structure of the peptide reveals that it belongs to the cystine knot inhibitor (ICK or knottin) family [4]. The ICK family contains numerous peptides and, as well as their related cyclized homologues (named cyclotides), they exhibit numerous biological activities presenting, potentially, a structural core for the design of new drugs or pesticides [5], [6], [7]. Using chemical synthesis and *in vitro* folding of PA1b [8], a collection of alanine mutants has been constructed. The three-dimensional structure of PA1b is particularly tolerant of any modifications. Receptor binding and insecticidal activities are both dependent on common well-defined clusters of residues located on one single face of the toxin, with Phe_10_, Ile_23_, and Leu_27_ as the key residues of the binding interaction corresponding to a hydrophobic area for bioactivity. An additional amino-acid, Arg_21_, represents a second strong area of interaction with the receptor [9].

The insecticidal activity of PA1b has been patented [1]. Indeed, PA1b has many attributes for use on an industrial scale: it is extracted from a commonly grown plant which is regularly consumed by humans and other mammals, it is suitable for use in transgenic plants, and it can withstand many stages of extraction and purification without losing its activity. Nevertheless, the current major disadvantage of PA1b is that it is only active at relatively high doses (for example, the dose used for routine laboratory tests on weevils is 400 µg/g of food). Hence, an important objective is to optimize the PA1b sequence in order to increase its activity towards insects. This research is based on the results of a structure/function study [9] and also on the natural diversity of A1b, found in a large number of Legumes [10]. The cDNA sequence of A1b isoforms from Fabaces, obtained from a gene or an EST bank, or by cloning in other Legumes, needs to be toxicologically tested on weevils in order to correlate the sequences with the activity.

To test these isoforms, it is necessary to produce them within a heterologous system. PA1b production in bacteria (*E. coli*) or yeast (*Pichia pastoris*) has never been successful. To date, only production by chemical synthesis and *in vitro* folding has enabled us to synthesize a biologically active PA1b. However, this system is not suitable for the expression of a large number of isoforms. Thus, we chose to investigate the expression of the toxin in a plant system, the tobacco plant *Nicotiana benthamiana*, with the aim of synthesizing and testing the activity of the six PA1b isoforms produced naturally by the pea, which cannot be purified by biochemical methods. Until now, the individual activities of each isoform have not been investigated.

## Results

### Expression of the Peptide(s) PA1b (and PA1a) in *N. benthamiana*

**Figure 2:**
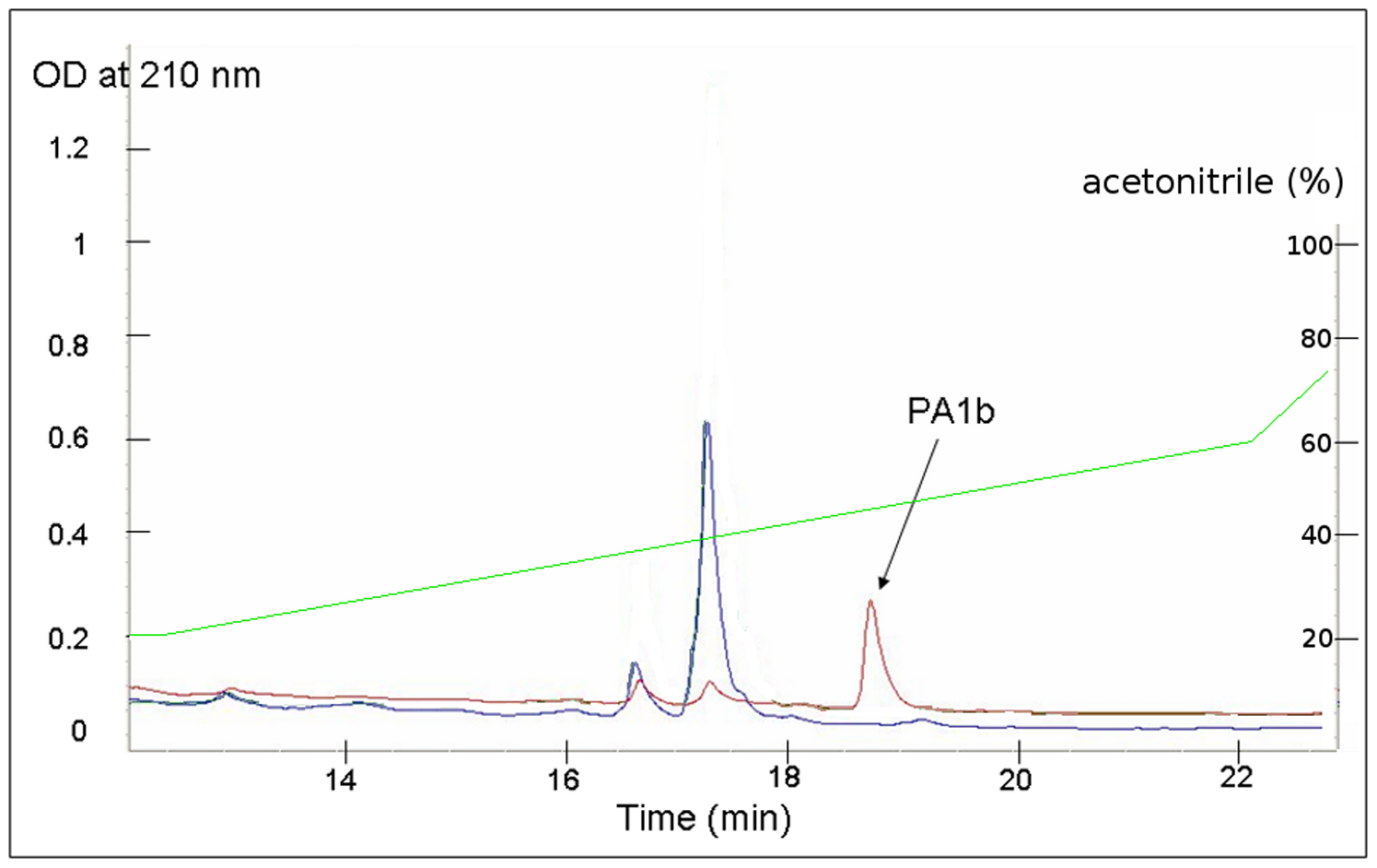
Production of PA1b in tobacco leaves. The figure shows the HPLC chromatograms of extracts from tobacco leaves infiltrated either with the p19 plasmid only (control, blue curve) or with p19 and the pMDC32 plasmid carrying the entire *PA1* cDNA (red curve). The figure shows the HPLC chromatograms of extracts from tobacco leaves infiltrated either with the p19 plasmid only (control, blue curve) or with p19 and the pMDC32 plasmid carrying the entire *PA1* cDNA (red curve). 5 days after infiltration, the leaves were harvested and, after partial purification, the mixture was injected into a C_18_ HPLC column and the elution was monitored at 210 nm. The green line represents the acetonitrile gradient.

The *PA1* cDNA from pea, coding for the signal peptide and the PA1 preproprotein, was inserted into the plant expression vector pMDC32. *A. tumefasciens* containing pMDC32 and *A. tumefasciens* containing the p19 plasmid (a suppressor of silencing) were co-infiltrated into *N. benthamania* leaves, and the inoculated leaves were harvested five days later. The HPLC chromatogram, presented in Figure 2, shows that the peptide PA1b was found in the pMDC32-(*PA1*cDNA) inoculated leaves but was not detectable in the control leaves inoculated with only the p19 plasmid. Using the chromatogram, we measured 21.4 µg of PA1b per g of fresh leaves. The mass spectrometry analysis corroborated the HPLC analysis: the only detectable peptide was PA1b in the pMDC32-(*PA1*cDNA) inoculated leaves extract, and the results confirmed that three disulfide bridges exist in the molecule. Nevertheless, three forms of the same peptide exist: the peptide with an additional Val_38_ (m/z = 3855.0), the pea native peptide (m/z = 3857.2) and, predominantly, the peptide lacking Gly_37_ (m/z = 3700.3). In addition, all forms have the oxidized Met_12_ peptide (+16 in mass). The PA1a peptide was not detectable on the HPLC chromatogram and the mass spectrometry analysis of the harvested leaves, at the 10–12 min elution time, displayed no m/z within the range of the PA1a mass.

To ensure that the expressed PA1b was in a biologically active form, we next performed toxicity assays on weevils (*S. oryzae*) and on Sf9 insect cells. The TL_50_ of the tobacco-expressed PA1b was similar to that of the same amount of pure PA1b peptide from pea seeds (7.8+/−1.13 and 7.23+/−1.1 days, respectively) on the WAA42 sensitive weevil strain. There was no mortality on the PA1b resistant strain ISOR3, confirming that the toxicity on the WAA42 strain was PA1b-like. Similarly, the LD_50_ values on Sf9 cells were 84+/−21 and 68+/−15 nM for the tobacco-expressed PA1b and for the PA1b from pea seeds, respectively.

### Kinetics of Expression of the Peptide PA1b in *N. benthamiana*

**Figure 3:**
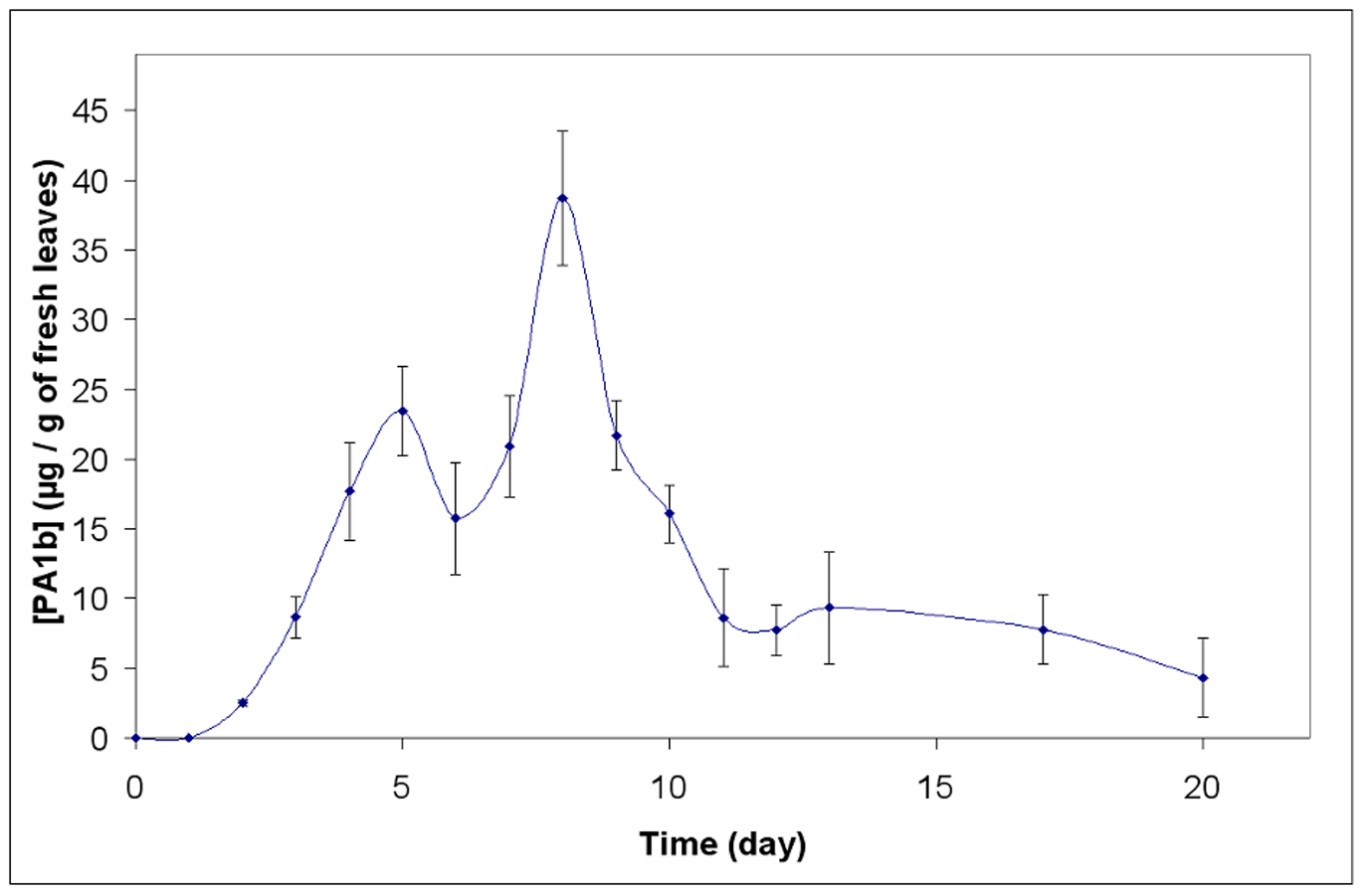
Kinetics of the expression of PA1b in tobacco. The data are the mean of three independent experiments. For each experiment, a set of 120 leaves were inoculated with the pMDC32 plasmid containing *PA1* cDNA. After extraction of the peptide from harvested leaves (5 leaves per day), the toxin was quantified by HPLC.

A kinetic analysis, to investigate the expression of PA1b in *N. benthamiana,* was carried out. The leaves were inoculated on day 0 and were harvested, over a range of time points, from day 1 to day 20 after inoculation. The PA1b and PA1a peptide were analyzed and quantified by HPLC. The results confirmed that the PA1a peptide was never detectable, and neither was the PA1b peptide in the control (p19) experiment. As regards the assay experiment (Figure 3), the PA1b peptide was visible two days after inoculation (2.5+/−0.2 µg/g of fresh leaves) and it increased to reach a maximum level 8 days after inoculation (38.7+/−4.8 µg/g of fresh leaves). Subsequently, the amount of PA1b found in the tobacco leaves decreased rapidly.

Mass spectrometry analysis of the PA1b produced in tobacco showed that, whilst 3 to 6 days after infiltration all three forms of the PA1b were present, 8 days after inoculation only one form was detectable (the peptide without Gly_37_ and with oxidized Met_12_, m/z = 3701.1).

In the next experiment, we harvested all the parts of the tobacco plant (i.e. transformed leaves, non-transformed leaves, stems and roots) on days 5, 8 and 12 after inoculation. Following extraction, the PA1b peptide was detected only in the transformed leaves and not in the other organs, using both HPLC and mass spectrometry.

### Role of the Different Parts of *PA1* cDNA on the Expression of PA1b

**Figure 4:**
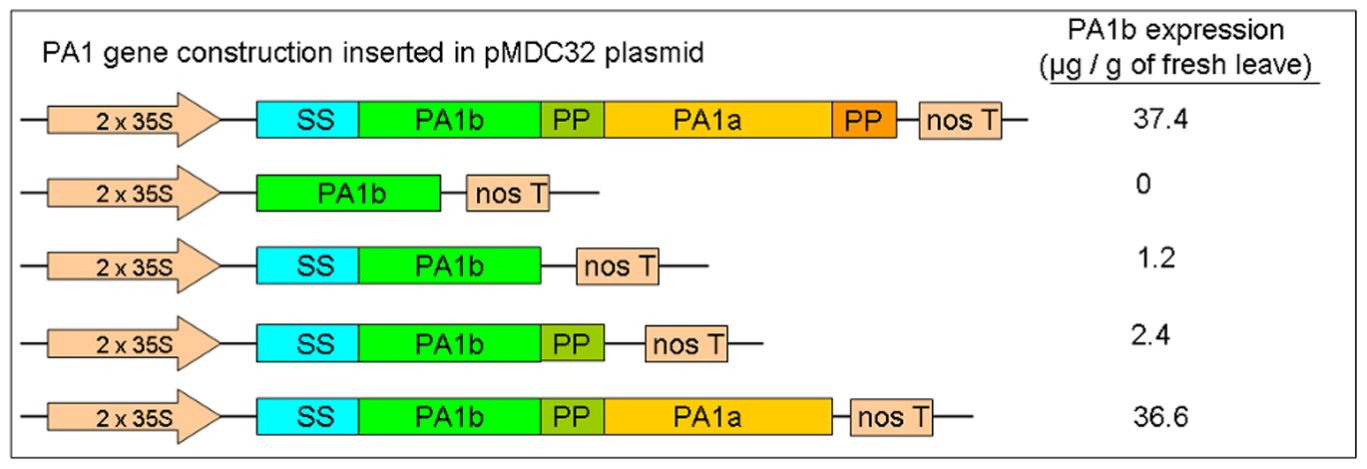
Different *PA1* constructions used for the expression of PA1b in *N. benthamiana*. The first (control) construction was the entire *PA1* cDNA. The other constructions were the cDNA coding PA1b alone; the signal sequence (SS) – PA1b; the SS – PA1b – propeptide(PP); and the SS – PA1b – PP – PA1a cDNAs. The *PA1* cDNA constructions were inserted in the pMDC32 plasmid under the control of the double CaMV35S promoter, and *Agrobacterium* were transformed with the different plasmids. After agro-infiltration of tobacco leaves, the PA1b expression, following HPLC purification, is indicated.

The relative importance of each part of the *PA1* cDNA was analyzed next. Different constructions of *PA1* cDNA were inserted into the pMDC32 plasmid (see Figure 4). Each modified plasmid was then introduced into *A. tumefasciens*, and the tobacco leaves were infiltrated. The results, presented in Figure 4, show that the amount of PA1b peptide extracted from tobacco leaves 8 days after inoculation was at a maximum level and was similar for the entire *PA1* cDNA and for the *PA1* cDNA lacking the terminal propeptide (37.4 and 36.6 µg PA1b/g of fresh leaves, respectively). In contrast, the constructions lacking the PA1a part of the gene showed a dramatic decrease in PA1b production (2.4 µg PA1b/g of fresh leaves). Finally, when only the *PA1b* cDNA was present, the peptide was undetectable by HPLC or by mass spectrometry.

### Construction of a PA1 Gene Cassette to Rapidly Express various PA1b Isoforms

Following our results, which indicated that the *PA1a* sequence is necessary for the production of the PA1b peptide, we next constructed a *PA1* cDNA allowing for the quick and easy replacement of the *PA1b* sequence by a modified one. First, two restriction sites, not present in the pMDC32 plasmid, were introduced by directed mutagenesis at the borders of the *PA1b* sequence (SnaBI and Acc651). This modified *PA1* sequence was expressed in tobacco: PA1b was expressed as for the native gene as regards the quantity (39.8 µg PA1b/g of fresh leaves) and the mass (m/z = 3700.9). The peptide was purified by HPLC and biological tests on weevils were then performed. The results showed that the toxin expressed from the *PA1*-cassette displayed a LD_50_ (8.41+/−1.42 days) similar to that of native PA1b on the WAA42 strain, and no mortality was observed on the ISOR3 resistant strain.

### Expression of Pea Peptides using the *PA1*-cassette

We next used the cassette system for expressing the peptides in tobacco plants and for biological testing of the others pea PA1b isoforms. The results, presented in Table 1, show that all the isoforms (numbered from 1 to 6) can be successfully produced in tobacco, with yields ranging from 17.61 to 53.33 µg/g of fresh leaves, except for isoform 3 (8.7 µg/g of fresh leaves). The mass spectrometry results showed that, in all the isoforms, the terminal Gly_37_ is lacking and the Met_12_ is oxidized (with the exception of isoform 2, where both forms were produced (numbered 2.1 and 2.2), and isoform 3, which was lacking the Met). Regarding biological activity, all isoforms have similar CL_50_ values on Sf9 insect cells (ranging from 53 to 99 nM) and similar TL_50_ values on the sensitive weevil strain WAA42 (ranging from 8.9+/−0.67 to 9.5+/−0.7 days), except for isoform 4 (269+/−50 nM on Sf9 and 12.9+/−1.1 days on weevils) and isoform 3 which was completely inactive on the Sf9 cells and on weevils. None of the isoforms had an effect on the resistant weevil strain ISOR3, confirming that this is a PA1b type of activity.

**Table 1:** Mass, sequences and biological activities of pea isoforms of PA1b.

Isoform number - Acc. number GenBank - Mass^1^	Yield^3^	CL_50_	5TL_50__WAA42_
Sequence^2^		Sf9^4^	TL_50 ISOR3_
1 - AJ574795–3701 Da	53.33	82 (24)	9.2+−0.9
ASCNGVCSPFEM_ox_PPCGSSACRCIPVGLLIGYCRNPS**G**			no mortality
2.1 - AJ574794–3750 Da	48.8	82 (36)	9.5+−0.7
ASCNGVCSPFEM_ox_PPCGTSACRCIPVGLFIGYCRNPS**G**			no mortality
2.2 - AJ574794–3734 Da	24.6	53 (16)	8.9+−0.6
ASCNGVCSPFEMPPCGTSACRCIPVGLFIGYCRNPS**G**			no mortality
3 - AJ574796–3732 Da	8.7	>1500	no mortality
ISCNGVCSPFDIPPCGSPLCRCIPAGLVIGNCRNPY **G**			no mortality
4 - M13790 and AJ276882*–3696 Da	41.95	269(55)	12.9+−1.1
ASCNGVCSPFEM_ox_PPCGSSACRCIPVGLVVGYCRHPS**G**			no mortality
5 - M13709–3688 Da	19.92	99 (31)	9.35+−0.7
ASCNGVCSPFEM_ox_PPCGTSACRCIPVGLVVGYCRNPS**G**			no mortality
6 - AJ574793–3700 Da	17.61	96 (22)	8.9+−0.7
ASCNGVCSPFEM_ox_PPCGTSACRCIPVGLVIGYCRNPS**G**			no mortality

^1^ Mass determined by MALDI-TOF analysis of the HPLC purified peptide.

_ox_ = oxidized Met. Differences from the first sequence are underlined. The amino-acids in bold were cleaved during the post-translational modifications.^2^ Peptidic sequences of the isoforms. M

µg per g of fresh leaves.^3^ In

^4^ In nM (SEM).

_50_ (days +/− SEM) on the sensitive weevil strain WAA42 (first line), and on the resistant strain ISOR3 (second line).^5^ TL

*PA1* cDNAs code for an identical PA1b peptide. The two

We next constructed a modified cDNA which encodes a peptide with two mutations: a Gly instead of the initial Ala_1_ and an Asn instead of the terminal Gly_37_. These modifications, in theory, produce a peptide which could be cyclized in the tobacco plants [11]. After purification, by HPLC, in the PA1b retention time range, the mass spectrometry results showed that only the linear peptide exists (m/z = 3670.2 and m/z = 3686.3 for the form with oxidized Met_12_). Based on measurement of the mass, this peptide is lacking the Asn_37_ terminal.

### Molecular Modeling of the Modified PA1b

**Figure 5:**
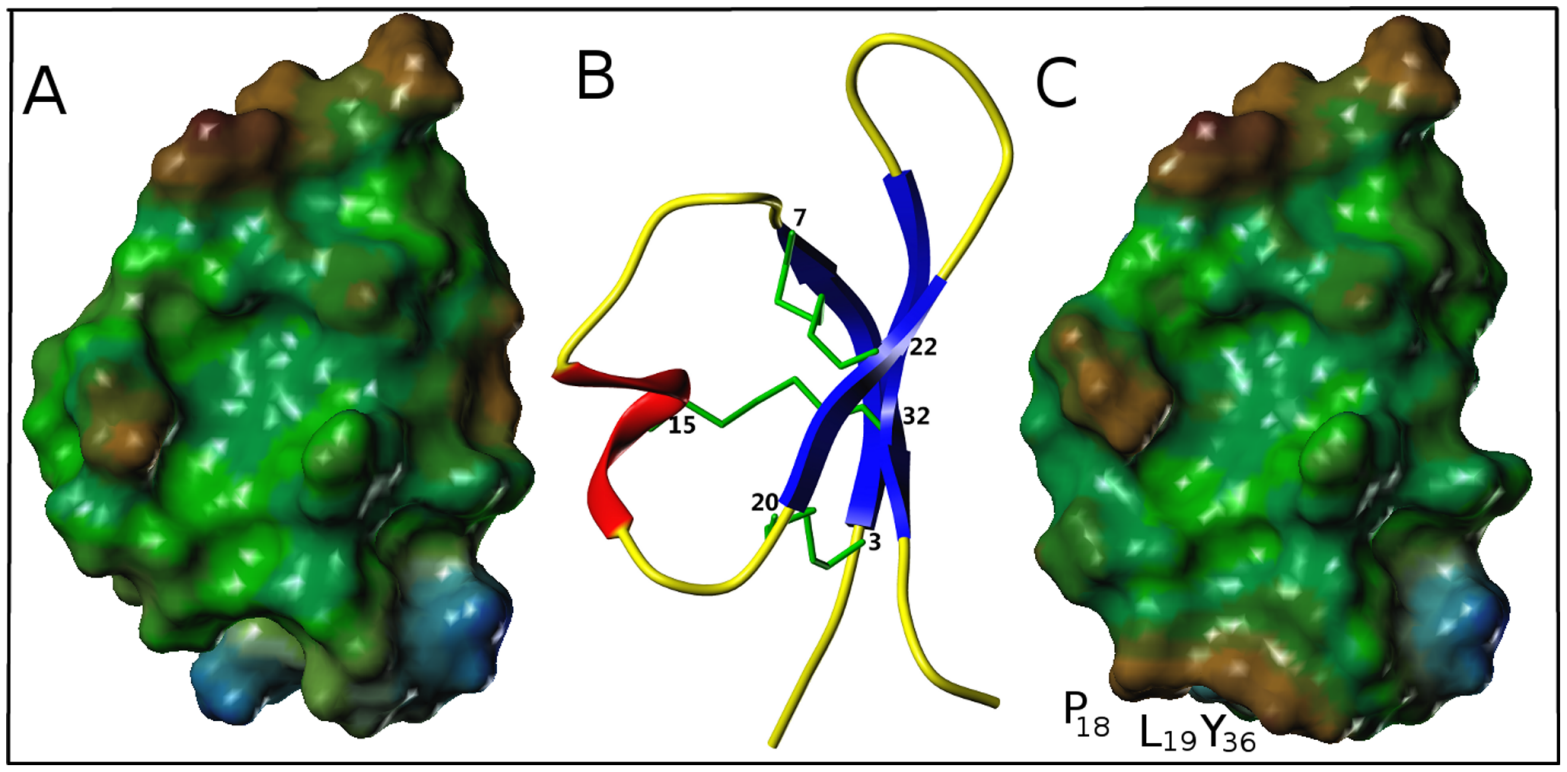
Lipophilic potentials of the PA1b isoforms 1 and 3. Lipophilic potentials, calculated with the MOLCAD option of SYBYL, at the Connolly surfaces of (A) PA1b isoform 1 and (C) isoform 3, and (B) ribbon representation of the PA1b isoform 1 NMR structure (the disulfide bridges are shown in green). Figures A and C are the same orientation as figure B, using a common hydrophobic scale. Hydrophobic and hydrophilic areas are displayed in brown and blue, respectively. Green surfaces represent an intermediate hydrophobicity.

The lipophilic potentials at the Connolly surfaces of isoforms 1 (control) and 3 were calculated. These molecular properties can be derived from the 3-D structures and are related to the functions of the proteins. The distribution of the hydrophobic potentials, calculated with MOLCAD at the surface of PA1b isoform 1, is characteristic of an amphipathic structure (Figure 5A). There is clearly a hydrophobic face formed by the residues of the hydrophobic loop (Val_25_, Leu_27_, Val_28_, and Ile_29_) together with residue Phe_10,_ which is opposite. The hydrophilic face, situated at the other pole of the molecule, arises from the following polar residues: Ser_2_, Asn_4_, Thr_17_, Ser_18_, Asn_34_, and Ser_36_. When the hydrophobic potentials are calculated, using the same hydrophobic scale, the surface of PA1b isoform 3 (Figure 5C) appears to be mainly hydrophobic, with a hydrophobic face replacing the hydrophilic face of PA1b due to the Pro_18_, Ile_19_, and Tyr_36_ mutated residues.

## Discussion

PA1b is an entomotoxin with considerable potential for applications on an industrial scale, the main advantage being that it is derived from an edible plant and, consequently, it can be used in organic farming. However, the main concern is the relatively high doses required for its activity. To overcome this problem, we have developed a program with the aim of determining the optimum PA1b sequence with maximum biological activity. This program is based on established data regarding the structure/function of the peptide [9] and on the existence of numerous isoforms in Legumes [10], [12]. Various peptide sequences are currently produced by chemical synthesis with *in vitro* folding [8]. However, this method is both expensive and long, with a variable yield depending on the isoforms. In this study, we succeeded, for the first time, in producing PA1b within a heterologous system, and we used this system to synthesize and test all the PA1b isoforms present in pea seeds.

In the extracts of pea seeds, the PA1b isoforms are always found in a unique form consisting of 37 amino acids (as indicated by mass spectrometry analysis). In tobacco plants, we extracted three forms of the peptide made up of 36, 37 or 38 amino acids. The cleavage of the proprotein seems to be different in tobacco, occurring after Val_38_ instead of after Gly_37_, as in the pea, indicated by the presence of the 38-mer peptide. After cleavage, the two terminal amino-acids are probably cleaved by a carboxypeptidase (not present in pea seeds), as indicated by a decrease in the 38-mer form which had been totally replaced, by day 8 after infiltration, by the 36-mer peptide. Similarly, replacing the Gly_37_ by an Asn results in a 36-mer, the Asn being cleaved in the same way as the Gly. Oxidation of Met_12_ is often found in PA1b, regarding the biological activity, we found that the lack of Gly_37_ and the oxidation of Met_12_ did not alter the toxicity of the peptide.

The kinetics of PA1b production in tobacco shows that production is at a maximum level on day 8, and it decreases rapidly thereafter. The optimum production time for other proteins is often earlier (days 4 to 6 after infiltration) [13], [14]. It has been demonstrated that the 3-D structure of PA1b (the cystine knot fold) confers a very high stability, especially regarding peptidase activities [4], [9]. This stability probably explains the increase in the production of PA1b right up to day 8. As PA1b has not been found in other plant organs (stem, root or leaves) on day 5, 8 or 13 after infiltration, we can hypothesize that PA1b is either ultimately degraded in the tobacco leaves by (induced?) proteases, or inactivated by coupling to another molecule.

Although, in theory, the two peptides PA1b and PA1a are produced in equimolar quantities, PA1a was never detected. No HPLC peaks were seen with a retention time corresponding to that of PA1a purified from pea seeds and even mass spectrometry, performed on the HPLC samples at the PA1a retention time, failed to detect any PA1a peptides. PA1b is a very stable peptide but this is not the case for PA1a, which has been shown to be less stable than PA1b in pea seeds [3]. Moreover, the 3D-structure of PA1a is much less organized than that of PA1b (L. Jouvensal and P. Da Silva, personal communication). It is thought that, in tobacco plants, PA1a has a very short half-life and is, therefore, not detectable.

We observed, in this study, that the entire cDNA is necessary for the expression of the PA1b peptide and that the presence of the PA1a sequence is also essential. These results raised the question of the role of PA1a, possibly at the level of RNA and involving a more stable *PA1* RNA rather than just the *PA1b* RNA alone. An alternative hypothesis of interest is that the PA1a peptide could act as a chaperone for the correct folding of PA1b. This activity could occur at the level of the proprotein (PA1a participating in the correct folding of the whole protein) or after the post-translational cleavage (PA1a perhaps assists in the folding of the PA1b peptide (and that of the PA1a itself?)). The importance of PA1a is supported by the recent discovery of a cyclotide (a cyclized cystine knot peptide called Cter M), in *Clitoria ternatea,* which is embedded in the *PA1* gene, i.e. the cyclotide sequence replaces the PA1b sequence in the *PA1* gene. Remarkably, this Cter M cyclotide displayed insecticidal activity against the cotton budworm *Helicoverpa armigera* [15].

The main objective of our study was to establish a quick and reliable expression system to test the many isoforms of the peptide for their toxicity, and to elucidate the effect of each isoform found in pea seeds. Nevertheless, although cloning and sequencing the PA1b part of the cDNA on various Legumes is relatively simple, cloning the entire *PA1* cDNA is often more complicated [10]. To simplify the expression of the PA1b isoforms, we constructed a PA1-cassette, with specific restriction sites bordering the PA1b part of the cDNA in order to easily change the PA1b-cDNA sequence whilst conserving the essential propeptides and PA1a sequences. The results demonstrate that this cassette allows for the expression of significant quantities of a biologically active toxin.

It has long been established that the mixture of isoforms in pea seeds is toxic for insects [1], [16], but the role of each individual isoform was not known due to the difficulty of separating these isoforms with HPLC or any other technique. Hence, our initial aim, using the *Nicotiana* system, was to produce all the pea seed isoforms in order to test them individually. The results show that all the isoforms have similar levels of activity, except for isoform 3 which was found to be inactive. This isoform is the one that differs most and three mutations (E_11_D, M_12_I and V_25_A) were previously found to have no, or only a slight, effect on activity [9]. Using molecular modeling, we found that three other mutations (S_18_P, A_19_L and S_36_Y) lead to the loss of the amphiphilic character of PA1b by changing the hydrophilic face to a hydrophobic one. This result completes the previous structure/function work [9]. The biological activity of PA1b depends on the presence of a hydrophilic area in the peptide (this work), and on the presence of the Arg_21_ residue and the hydrophobic nature of the PA1b loop opposite the hydrophilic loop [9]. Moreover, the replacement of Leu28 by a Phe, in isoform 2, confirms that the overall hydrophobicity of this loop is more important than the nature of the amino acid side chain.

The cyclization of a peptide only occurs rarely, but this leads to a very stable structure [17] and we suggest that a cyclized PA1b may show stronger activity. Moreover, cyclotides have been proven to be useful scaffolds for stabilizing bioactive peptides [18]. The cyclization of PA1b could help to identify new insecticides. It has been demonstrated that peptide cyclization depends on an enzyme, asparaginyl endopeptidase, which is present in tobacco [19]. In addition, this cyclization depends on the peptide sequence, and the presence of a C terminal Asn and an initial Gly were found to be very important [11], [20]. For this reason, the A_1_G and G_37_N mutations were inserted in the PA1b sequence, but this did not result in the production of cyclic PA1b. The linear peptide produced by the tobacco plant lacked the terminal, and essential, Asn. It is difficult to establish whether cyclization does not occur because of the proteolysis of Asn or because PA1b cannot be cyclized in tobacco. Studies on the cyclization of peptides have been conducted on naturally cyclized peptides, the cyclotides [6], [17]. PA1b may be more difficult to cyclize because it is longer (37 vs. 29–30 amino acids) and its primary sequence is very different from those of cyclotides, although it has the same cystine knot fold.

However, this work enabled us to easily express a sulfur-rich plant peptide in tobacco, which was previously shown to be not feasible in *E. coli* or in *P. pastoris*. Also, the entire *PA1* gene is shown to be necessary for effective expression and we constructed a PA1-cassette for the quick and reliable expression of a large number of PA1b peptides. Subsequently, in our study, this cassette system expression allowed us to determine the real effect of each pea isoform, providing important information about their structure/function. Such an expression system will help in determining the best sequence for producing the most efficient toxin.

PA1b belongs to the ICK family, which contains numerous peptides with various biological activities [7]. This cystine knot core is increasingly considered to be a valuable structural platform for creating compounds with numerous biological targets, particularly in the field of therapeutics and of agriculture, and the PA1b-cassette expression could be useful in other research involving ICK peptides. Until now, ICK peptides have been produced either by a heterologous system or by chemical synthesis. The success of production in *E. coli* depends greatly on the peptide and, for chemical synthesis, the limiting step is often the folding of the cystine knot and its three disulfide bridges [21]. It is possible that our expression system could provide a means of producing cystine knot peptides which are otherwise difficult to produce, in *E. coli* or by chemical synthesis, as well as a quick and valuable tool for any structure/function studies.

## Materials and Methods

### *N. benthamiana* Culture

*Nicotiana* plants were grown in a greenhouse with a 16 h-day photoperiod, at a controlled temperature of 24°C (day) or 21°C (night). 8 to 10 week-old plants were chosen for *Agrobacterium* infiltration.

### PA1b Extraction, Identification and Quantification

Tobacco leaves were harvested and immediately ground, in a mortar, in liquid nitrogen. The resulting powder was incubated in 10 volumes of 60% ethanol for 2 h, at 4°C. Next, the mixture was centrifuged at 10 000 g for 10 min and the ethanol was evaporated under vacuum. The remaining solution was dried by lyophilisation and the powder was stored at −20°C until required.

The powder was re-suspended in 60% EtOH (10 mg.mL^−1^) and injected onto a reverse-phase HPLC column (Jupiter C18 5 µm, 300 A, 250×4.6 mm, Phenomenex), in an Agilent 1200 apparatus with elution, as described in [22]. The peptides were harvested according to their retention times (typically between 18 and 20 min for PA1b and between 10 and 12 min for PA1a) and the samples were then lyophilised. PA1b was quantified, using HPLC, by comparison with known quantities of pure PA1b from pea seeds.

Peptides were identified by mass spectrometry. The samples were analyzed on a MALDI-TOF MS (Autoflex, Bruker Daltonics), operated in reflectron positive mode.

### Toxicity Assays

Rice weevils (*Sitophilus oryzae*, Coleoptera) were reared on wheat seeds at 27.5°C and 70% RH. Tests and survival analysis were performed on adults feeding on food pellets (composed of wheat flour and water) incorporating PA1b (extracted from pea seeds for control tests or produced in tobacco) at 400 µg.g^−1^, as described in detail in [10]. LT_50_ values were calculated using the SIMFIT software (http://www.simfit.man.ac.uk). The PA1b resistant strain, ISOR3, was reared in an identical way except that the weevils were fed on pea seeds rather than on wheat seeds.

*Spodoptera frugiperda* Sf9 cells were grown and used for viability assays, according to [23].

### PCR Conditions

The mixture for PCR (20 µl) consists of 2 µL of reaction buffer 10X (670 mM Tris-HCl, pH8.8, 160 mM (NH_4_)_2_SO_4_, 0.1% Tween-20) (Ozyme), 1 µl of 50 mM MgCl_2_, 1 µL of 10 mM dNTP, 1 µL of 1 µM forward and reverse primers, 2 µL of DNA template and 0.25 µL of 5 U.µl^−1^ high fidelity Taq-polymerase (Platinium Taq DNA polymerase High fidelity, Invitrogen), and PCR assays were performed using a BIO-Rad C1000 thermal cycler.

The PCR conditions were 5 min at 95°C, then 30 cycles of [95°C, 30 sec/50°C, 1 min/72°C, 1 min] and, finally, 72°C for 10 min.

For directed mutagenesis, the PCR conditions were 95°C for 5 min, then 28 cycles of [95°C, 30 sec/50°C, 1 min/72°C, 1 min] for the first and second PCR whereas, for the third PCR, we used 95°C for 5 min, then 30 cycles of [95°C, 30 sec/50°C, 1 min/72°C, 1 min] and 72°C, for 10 min.

### Construction of the PA1 and PA1 Truncated Variants Expression Plasmid

The sequences of all the primers used in this paper are given in the Table S1 and were synthesized by Eurogentec (France). The cDNA used for PCR corresponds to the pea gene with accession number AJ574795.

The entire *PA1* cDNA codes for a peptide with a signal sequence (SS), the PA1b peptide, an initial propeptide (PP), the PA1a peptide, and a second propeptide (PP).

The entire *PA1* cDNA (SS-PA1b-PP-PA1a-PP) was amplified, by PCR, with two specific primers (Sac1PA1F and BamH1PA1R) to incorporate the enzyme restriction sites Sac1 and BamH1. In the same way, the SS-PA1b-PP-PA1a, the SS-PA1b-PP, the SS-PA1b, and the PA1b (with inclusion of a start codon) cDNAs were amplified with, respectively, the specific primers Sac1PA1F and BamH1PA1R, BamH1PA1R and Sac1PA1-P3F; BamH1PA1R and Sac1PA1b+P2F; BamH1PA1R and Sac1PA1bF; and BamH1PA1-P1 and Sac1PA1bF. Next, they were sub-cloned into the pCR2.1 vector (TOPO TA Cloning, Invitrogen). With enzyme BamH1/Sac1 restriction cutting, *PA1* cDNA and the truncated variants were inserted into the pMDC32 vector, provided by http://www.arabidopsis.org/abrc/catalog/vector_1.html [24], between the cauliflower mosaic virus (CaMV) double 35S constitutive promoter and the nos transcriptional termination region.

For the pea gene (with the intron), the primers used for PCR amplification were the same as for the entire *PA1* cDNA and genomic DNA from *P. sativum* was used as a template.

### Transformation of *N. benthamiana*

*Agrobacterium* C58pMP90 cells, carrying the pMDC32 plasmid with *PA1* cDNA or *PA1* truncated variants, and *Agrobacterium* carrying the p19 plasmid [25] were grown in Luria Broth (LB) media, containing 50 µg.ml^−1^ kanamycin and 50 µg.ml^−1^ rifampicin, at 28°C. When the A_600_ reached 0.6 the cells were pelleted (3500 g for 10 min, room temperature) and then twice re-suspended in an equal volume of MES buffer (50 mM MES pH 5.6, 2 mM NaH_2_PO_4_, 100 µM acetosyringone, 0.5% glucose). Each suspension of *Agrobacterium,* carrying pMDC32 with a different *PA1* construct, was incubated with the suspension of *Agrobacterium* carrying p19 (50/50), at room temperature for 1–2 h, and then infiltrated into *N. benthamiana* leaves, following the methods of [26], [27].

### Construction of the *PA1*-cassette

Two new enzyme restriction sites (SnaBI and Acc651) were inserted into the pMDC32– *PA1* vector, framing the PA1b sequence in codons 23 and 69, respectively. Mutagenesis was carried out, by PCR, using oligonucleotides containing the required mutations. To generate the necessary site-directed mutagenesis constructs, a three-round asymmetric PCR strategy was used for each enzyme restriction site, as previously described [28]. The first two reactions were carried out using the pMDC32-*PA1* vector as the DNA template, and for the SnaBI restriction site mutation, respectively, primers BamH1PA1R and SnaBIPA1R, and Sac1PA1 F and SnaBIPA1F were used. The two resulting DNA products were purified and used as templates for the third PCR, with primers Sac1PA1F and BamH1PA1R. This new PA1-SnaBI was inserted into the pCR2.1 plasmid and used as a template for the insertion of the Acc651 enzyme restriction site. Following the same pattern, the Acc651 mutation was inserted, using a three-step PCR, with the following primer couples: PA1Acc651F and PA1BamH1R; Sac1PA1F and PA1Acc651R; PA1BamH1R and Sac1PA1F. This new sequence was then inserted, by enzymatic cutting (BamH1, Sac1), into the pMDC32 vector.

### Insertion of PA1b Pea Isoforms into a *PA1*-cassette

The mutated sequences of PA1b were obtained from a pMDC32 *PA1*-cassette by PCR, using specific long primers as described in S1. The newly created sequences were cloned in pCR2.1 and were inserted into the pMDC32-*PA1* cassette by SnaBI and Acc651 enzymatic cutting. *N. benthamiana* leaves were transformed and toxicity tests were carried out as explained above.

### DNA Quantification and Sequencing

DNA was quantified using a NanoDrop ND-1000 spectrophotometer and DNA sequences were performed by BioFidal (Vaux-en-Velin, France).

### Sequence Alignments and Comparative Modeling

Sequence alignments were performed using CLUSTAL OMEGA [29]. Comparative modeling was performed using the ORCHESTRAR homology modeling program in the SYBYL-X 2.0 software package (TRIPOS Inc., St Louis, MO). The PA1b NMR structure (PDB code: 1P8b) was used as a template to build the 3D structure model of the PA1b isoforms. Lipophilic potentials were calculated and represented using the MOLCAD option of the SYBYL-X 2.0 software package.

## Supporting Information

Table S1 **Sequences of the primers used in this paper.**
(DOC)
