# Supplementary material for: Expression and Biological Activity of the Cystine Knot Bioinsecticide PA1b (Pea Albumin 1 Subunit b)
Source: PLoS One. 2013 Dec 11;8(12):e81619. doi: 10.1371/journal.pone.0081619 (PMC3859497; doi:10.1371/journal.pone.0081619)
Supplement: Table S1 — Sequences of the primers used in this paper. (DOC) [file pone.0081619.s001.doc]

**Table S1.** Sequences of the primers used in this paper.

| Amplified cDNA | Primers couple | Sequence of the primers |
| --- | --- | --- |
| **Primers for the construction of the PA1 and PA1 truncated variants expression plasmid** | | |
| *PA1* cDNA (SS-PA1b-PP-PA1a-PP) | Sac1PA1F BamH1PA1R | 5’ATGAGCTCTTAAGCAGTGGAAACACTC 3’  5’ ATTGGATCCATGGCTTCCGTT 3’ |
| PA1b | PA1bF Sac 1  PA1bR BamH1 | 5’ATGAGCTCTTATCCAGATGGATTTCTGC 3’  5’ AAGGATCCATGGCAAGCTGCAATGGG 3’ |
| SS-PA1b | PA1bF Sac 1  PA1R BamH1 | 5’ATGAGCTCTTATCCAGATGGATTTCTGC 3’  5’ATTGGATCCATGGCTTCCGTT 3’ |
| SS-PA1b-PP | PA1bF+pp1 Sac1  PA1R BamH1 | 5’CTGAGCTCTTAATTCCCCTTCAAGAAAACTCC 3’  5’ATTGGATCCATGGCTTCCGTT 3’ |
| SS-PA1b-PP-PA1a | PA1aF Sac1  PA1R BamH1 | 5’CTGAGCTCTTAGTCTTTTGGGGTAATCTTAG 3’  5’ATTGGATCCATGGCTTCCGTT 3’ |
| **Primers for the directed mutagenesis** | | |
| PCR 1 SnaB1 | SnaB1PA1F BamH1PA1R | 5’ GCAGCTTGCTGCTCCTACGTATTT 3’  5’ ATTGGATCCATGGCTTCCGTT 3’ |
| PCR 2 SnaB1 | SnaB1PA1R Sac1PA1 F | 5’ AAATACGTAGGAGCAGCAAGCTGC 3’  5’ATGAGCTCTTAAGCAGTGGAAACACTC 3’ |
| PCR 3 SnaB1 | Sac1PA1F BamH1PA1R | 5’ATGAGCTCTTAAGCAGTGGAAACACTC 3’  5’ ATTGGATCCATGGCTTCCGTT 3’ |
| PCR 1 Acc651 | PA1Acc651F PA1BamH1R | 5’ GTTCATCGGTACCCTTCAAGAA 3’  5’ ATTGGATCCATGGCTTCCGTT 3’ |
| PCR 2 Acc651 | Sac1PA1F PA1Acc651R | 5’ TTCTTGAAGGGTACCGATGAAC 3’  5’ATGAGCTCTTAAGCAGTGGAAACACTC 3’ |
| PCR 3 Acc651 | PA1BamH1R Sac1PA1F | 5’ GTTCATCGGTACCCTTCAAGAA 3’  5’ ATTGGATCCATGGCTTCCGTT 3’ |
| **Primers for the insertion of pea isoformes cDNA in the PA1-cassatte** | | |
| PA1b isoformes coded by the pea gene M13709 | PA1b K7 2F  PA1b K7 2R | 5’TCGGTACCCCTCAAGAAAACTCCAGATGGATTTCTGCAGTAACCAATAACT 3’  5’AtACGTAGGAGCAGCAAGCTGCAATGGGGTTTGTTCTCCATTTGAGATGCCACCATGTGGCACTTCAGCTT 3’ |
| PA1b isoformes coded by the pea gene AJ574794 | PA1b K7 2F  PA1b K7 3R | 5’TCGGTACCCTTCAAGAAAACTCCAGATGGATTTCTGCAGTAACCTATAA 3’  5’AtACGTAGGAGCAGCAAGCTGCAATGGGGTTTGTTCTCCATTTGAGATGCCACCATGTGGCACTTCAGCTT 3’ |
| PA1b isoformes coded by the pea gene AJ574796 | PA1b K7 5F  PA1b K7 5R | 5’TCggTaccCCTCAAGAAAACTCCATATGGATTTCTGCAGTTACCAATAACTAGACCAGCA 3’  5’AtACGTAGGAGCAATAAGCTGCAATGGGGTTTGTTCTCCATTTGATATCCCACCATGTGGCTCTCCACTTT 3’ |
| PA1b isoformes coded by the pea gene AJ574793 | PA1b K7 2F  PA1 K7 6R | 5’TCGGTACCCCTCAAGAAAACTCCAGATGGATTTCTGCAGTAACCAACAACTA 3’  5’ATACGTAGGAGCAGCAAGCTGCAATGGGGTTTGTTCTCCATTTGAGATGCCACCATGTGGCACTTCAGCTT 3’ |
| PA1b isoformes coded by the pea gene AJ276882 | PA1b K7 7F  PA1b K7 7R | 5’ATCGGTACCCCTCAAGAAAACTCCAGATGGATGTCTGCAGTAACCAACAACTAG 3’  5’AATACGTAGGAGCAGCAAGCTGCAATGGGGTTTGTTCTCCATTTGAGATGCCACCATGTGGCTCTTCAGCCTGTC 3’ |
| PA1b cyclization | PA1b CRK3 K7 F  PA1b CRK3 K7 R | 5’ CCTGACAAAATACGTAGGAGCAGGAAG 3’  5’ TCATCGGTACCCTTCAAGAGAGCGTTAG 3’ |
